# Genomic Imprinting in Mammals: Emerging Themes and Established Theories

**DOI:** 10.1371/journal.pgen.0020147

**Published:** 2006-11-24

**Authors:** Andrew J Wood, Rebecca J Oakey

**Affiliations:** Massachusetts General Hospital, United States of America

## Abstract

The epigenetic events that occur during the development of the mammalian embryo are essential for correct gene expression and cell-lineage determination. Imprinted genes are expressed from only one parental allele due to differential epigenetic marks that are established during gametogenesis. Several theories have been proposed to explain the role that genomic imprinting has played over the course of mammalian evolution, but at present it is not clear if a single hypothesis can fully account for the diversity of roles that imprinted genes play. In this review, we discuss efforts to define the extent of imprinting in the mouse genome, and suggest that different imprinted loci may have been wrought by distinct evolutionary forces. We focus on a group of small imprinted domains, which consist of paternally expressed genes embedded within introns of multiexonic transcripts, to discuss the evolution of imprinting at these loci.

## Introduction

The process of sexual reproduction dictates that mammals inherit two copies of every gene, one from the mother and one from the father. At most loci, both alleles are actively transcribed and functionally equivalent. Imprinted genes represent an exception to this rule, as the transcriptional activity of each allele is determined by the gender of the parental germ line to which it was most recently exposed. This parental legacy is initiated by epigenetic modifications such as DNA methylation, which is established in the parental germ line and maintained throughout somatic development in the offspring. Individual germ-line marks can control the allele-specific silencing or activation of multiple neighbouring genes, which leads in many instances to clusters of imprinted transcripts. Such loci represent an attractive paradigm for the study of epigenetic transcriptional regulation, as both the active and silent allele are present in the same cell nucleus, and therefore potentially exposed to the same *trans*-acting regulatory factors. Epigenetic abnormalities at imprinted loci have been observed in cloned mammals [1], and their disruption has been reported in a number of human developmental disorders and cancers [2].

## Defining the Extent of Imprinting

Since the identification of the first autosomal imprinted genes in the early 1990s [3–5], much speculation has surrounded the question of how many exist. Attempts to count the exact number have been complicated by difficulties in defining exactly what constitutes a gene, as in several cases multiple functional components are derived from a single core of genetic information [6]. A recent census identified 96 imprinted functional components (54 maternally expressed, 42 paternally expressed) arising from 71 transcriptional units [7], and the relevant literature is summarised on the Harwell and University of Otago online databases [8,9].

A number of different approaches have been employed to define the extent of imprinting in the mouse genome. Mouse stocks carrying translocation chromosomes were used to define chromosomal regions that show parent-of-origin effects on phenotype when uniparentally inherited, and at least 13 distinct regions on eight chromosomes have been identified by this approach (C. V. Beechey, personal communication; [8]). The phenotypes range from early embryonic lethality to postnatal effects on growth and development, and are likely to result from the misexpression of imprinted genes situated within the uniparentally duplicated region [10]. The subsequent identification of imprinted genes on chromosomes without obvious uniparental effects [11–13] suggests that imprinting may be more widespread than initially thought, and not limited to genes that are vital for development. This conclusion is supported by the involvement of imprinted genes in behavioural traits in the mouse [14,15].

A number of more recent technologies have identified imprinted genes, and these are covered in detail elsewhere [16–18]. Early estimates put the total number of imprinted loci between 100 and 200 [19,20]. A more recent study based on sequence features in the region of known imprinted promoters identified 600 genes that are potentially imprinted [21]. This survey is a valuable resource, particularly when used in combination with expression screens, but this informatic approach suffers from several drawbacks. A number of genes undergo imprinting as a result of epigenetic modifications established on sequences that are situated several hundred kilobases away [22,23]; hence, the regions flanking their promoters may not directly provide the information required for imprinting. Some imprinted genes exhibit monoallelic expression in a limited number of cell lineages, and therefore the verification of these 600 candidates is problematic without information on tissue specificity. If tissue-specific imprinting is a common theme, as seems to be the case [24], then the number of known imprinted genes in the mouse is likely to increase substantially.

**Figure 1:**
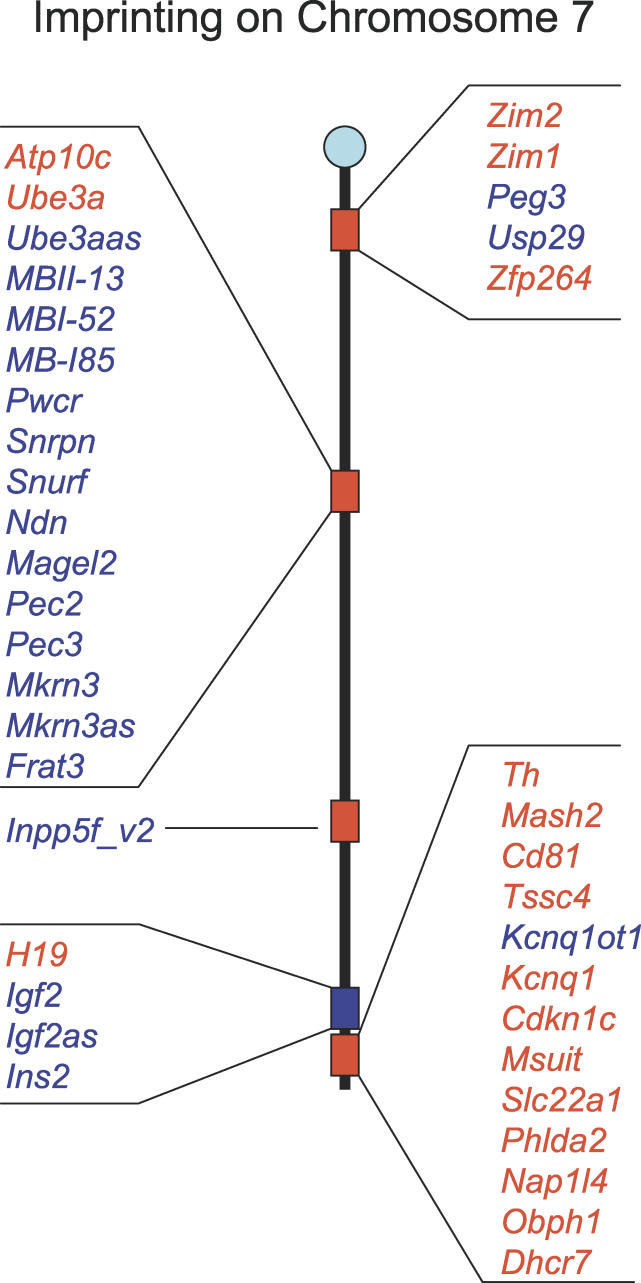
Imprinting on Mouse Chromosome 7 Maternally expressed genes are shown in red, paternally expressed in blue. Individual clusters are controlled by oocyte-derived (red) or sperm-derived (blue) methylation marks.

Imprinted genes that have been identified in the mouse are distributed unevenly throughout the genome [8]. Approximately half of the total number is situated on Chromosome 7, clustered into at least five distinct imprinted domains (Figure 1 and [25–28]). Additional genome-wide screens will help determine whether this reflects a sampling bias in the methods that have been employed to identify these genes, or a genuine predisposition to imprinting in certain genomic regions.

**Figure 2:**
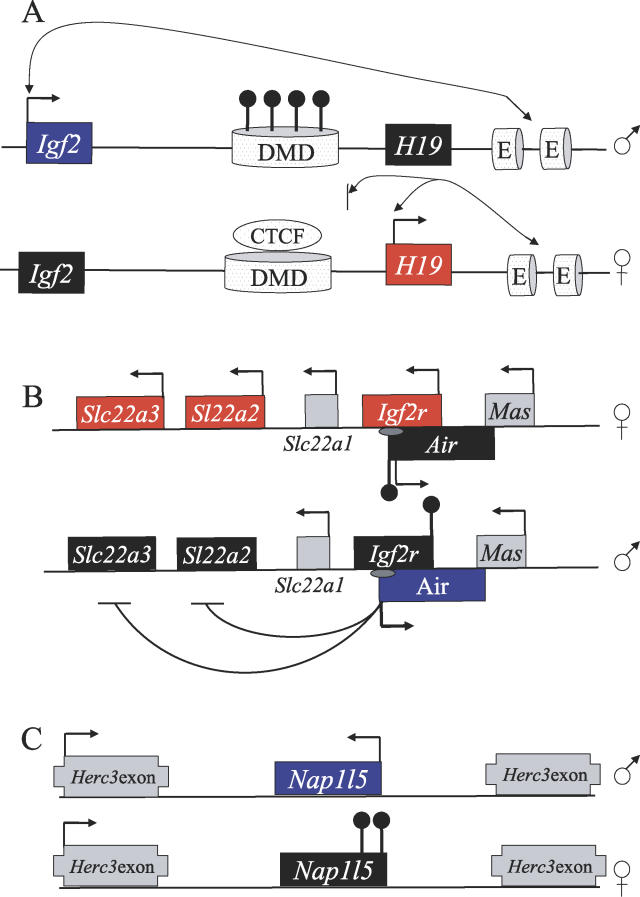
Regulatory Models at Imprinted Loci Blue boxes represent paternally expressed alleles, red boxes maternally expressed alleles, black boxes silenced alleles, and grey boxes nonimprinted genes. Arrows on boxes indicate transcriptional orientation. (A) The enhancer–blocker model (also known as the boundary model) is well studied at the *Igf2/H19* locus and consists of an ICR located between a pair of reciprocally expressed genes that controls access to shared enhancer elements [38,116]. On the paternal allele, the differentially methylated domain (DMD) acquires methylation (black circles) during spermatogenesis, which leads to repression of the *H19* promoter [117]. The hypomethylated maternal DMD acts as an insulator element, mediated through binding sites for the methylation-sensitive boundary factor CTCF (shaded ellipse). When CTCF is bound, *Igf2* promoter access to the enhancers (E) distal to *H19* is blocked. (B) At the *Igf2r* locus on Chromosome 17, the paternally expressed, noncoding RNA *Air* acts to induce bidirectional *cis*-mediated silencing (black curved lines) on neighbouring protein-coding genes (maternally expressed *Igf2r, Slc22a3,* and *Slc22a2*) [50]. The grey ellipses are the intronic imprint control elements that are maternally methylated (black circles) and contain the promoter of the *Air* RNA. (C) At microimprinted domains, oocyte-derived methylation in the promoter region of a protein-coding gene is likely to be the primary epigenetic mark leading to monoallelic silencing. With the exception of the *U2af1-rs1* locus, the multiexonic genes within which the paternally expressed transcripts are embedded, escape imprinting (Table 1). The paternally expressed *Nap1l5* is situated within intron 22 of *Herc3,* which is expressed from both alleles.

Imprinted genes can occur in isolation but are frequently found in clusters that share common *cis*-regulatory elements that can act over distances of a megabase or more [23]. The DNA sequences responsible for the establishment of imprinting are termed imprinting control regions (ICRs) and undergo differential patterns of methylation at CpG dinucleotides when passing through the maternal and paternal germ line [29,30]. In some instances, these epigenetic marks act after fertilisation to mediate the establishment of additional marks at adjacent loci, conferring imprinting on neighbouring genes [31]. As an increasing number of imprinted chromosomal domains have been studied, it has become clear that monoallelic expression is achieved in different ways at different loci. A large body of work has focused on dissecting the mechanisms by which this coordinate epigenetic regulation is achieved, and this topic has been the subject of a number of reviews in recent years [32,33]. Some of the well-characterised regulatory models are illustrated in Figure 2.

**Table 1:**
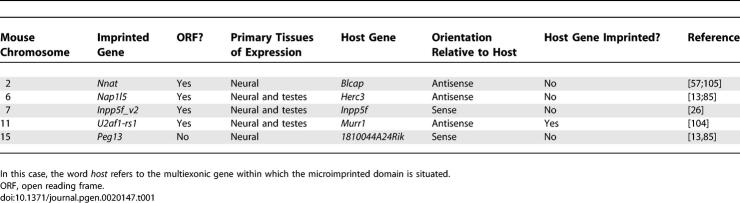
Properties of the Five Known Microimprinted Domains in the Mouse.

## Trends and Mechanisms

The paternally methylated ICR at *Igf2*/*H19* is situated several kilobases upstream of the *H19* gene [34], and this intergenic location is seen at the other two known paternally methylated ICRs at *Dlk1*/*Gtl2* [35] and *Rasgrf1*/*A19* [36,37]. The CCCTC-binding factor (CTCF) mediates methylation-sensitive insulator activity on the unmethylated maternal allele for both the *Igf2* [38] and *Rasgrf1* [39] ICRs, and has also been shown to bind at the human *DLK1/GTL2* locus [40]. These paternally methylated sequences may therefore commonly act as insulators on the unmethylated maternal allele. Recent work at the *Igf2* locus has highlighted the importance of intrachromosomal and interchromosomal chromatin structure facilitating interactions between regulatory regions [41,42].

Although the total number of maternally and paternally expressed genes is approximately even, differences exist in the proportion of those controlled by maternal and paternal methylation marks. Oocyte-derived methylation marks at imprinted regions are overrepresented relative to their paternal counterparts, and it has been suggested that this may result from the active and widespread demethylation of the paternal pronuclear genome that occurs following fertilisation in several mammalian species [43–45]. Although germ-line methylation marks have not yet been identified for all of the known imprinted loci, of the 15 that have been found, 12 are of maternal origin, while only three are paternally derived [29,30,46–49].

All of the sequences associated with imprinted loci that are known to undergo methylation during oogenesis also possess promoter activity on the unmethylated paternal allele, and at least three of these promoters give rise to noncoding RNAs and can confer epigenetic silencing on neighbouring genes in *cis* [22,50–52] (Figure 2). These and other sexually dimorphic trends in the nature of ICR function have been discussed in [53,54].

## Microimprinted Domains

DNA methylation marks of maternal germ-line origin are also seen at microimprinted domains, a term used to describe paternally expressed transcripts with few or no introns that are situated entirely within introns of other genes (Figure 2 and Table 1). Here, maternal germ-line methylation in the region of a promoter brings about highly localised silencing on the maternal allele by facilitating the formation of repressive chromatin structures [55,56]. The minimal nature of this mechanism has led to the proposal that it represents a primordial imprint, and that additional complexity may evolve over evolutionary time [54,57]. The observation that at least four of the five known microimprinted domains arose specifically in eutherian mammals appears to support these ideas ([57,58]; A. J. Wood, unpublished data), and three of this number *(Inpp5f_v2, U2af1-rs1,* and *Nap1l5)* bear the hallmarks of retrotransposition.

Randomly inserted transgenes carrying retroviral and bacterial sequences can also undergo differential patterns of gametic methylation [59,60], and the site of integration is critical in determining whether the inserted sequences are imprinted [60]. Retrogenes can be considered as naturally occurring transgenes, and appear to be subject to similar types of position-dependent epigenetic effects at the site of integration. In contrast, transgenes carrying endogenous ICR sequences and their flanking regions can undergo imprinting regardless of their chromosomal location [61,62]. The targeted deletion of specific sequences within these transgenes indicates that the sequences flanking the differentially methylated regions were necessary for the consistent establishment of imprinted methylation [62]. The sequences flanking ICRs often consist of short direct repeats, which might act to guide the establishment of CpG methylation in the germ line (see below).

## Molecular Events and Selective Forces

**Figure 3:**
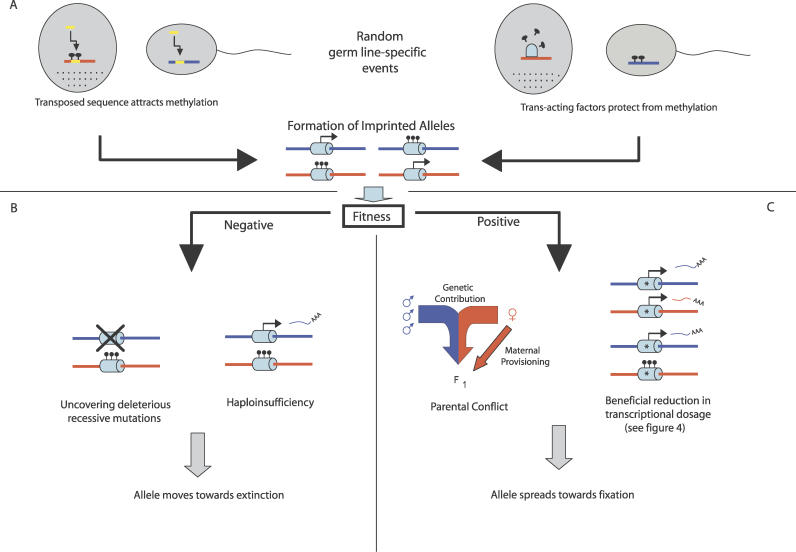
Overview of the Evolution of Imprinted Loci Blue represents paternal or paternally derived alleles, red represents maternal or maternally derived alleles, and yellow represents transposed sequence. Black lollipops represent methylated CpGs, and the light blue dome represents a *trans*-acting factor. An asterisk denotes a gene duplicate. (A) Random molecular events or mutations in the germ-cell lineage generate alleles that undergo differential methylation when passing through the male and female germ line, which can confer either (B) negative or (C) positive fitness. While most of these alleles would be expected to confer negative fitness (B), a small proportion are maintained (C). Possible reasons for the spread of these alleles (C) are discussed further in the text and in Figure 4, which are by no means intended to be exhaustive. F1, first filial generation.

The process of evolution involves Darwinian selection acting on random molecular events such that when new alleles are generated that confer positive fitness, they are preferentially maintained in a population. For a complete understanding of the evolution of genomic imprinting, it is therefore necessary to consider both the molecular events that generate novel imprinted alleles and the selective forces that might act to maintain those epialleles [63] (Figure 3).

**Figure 4:**
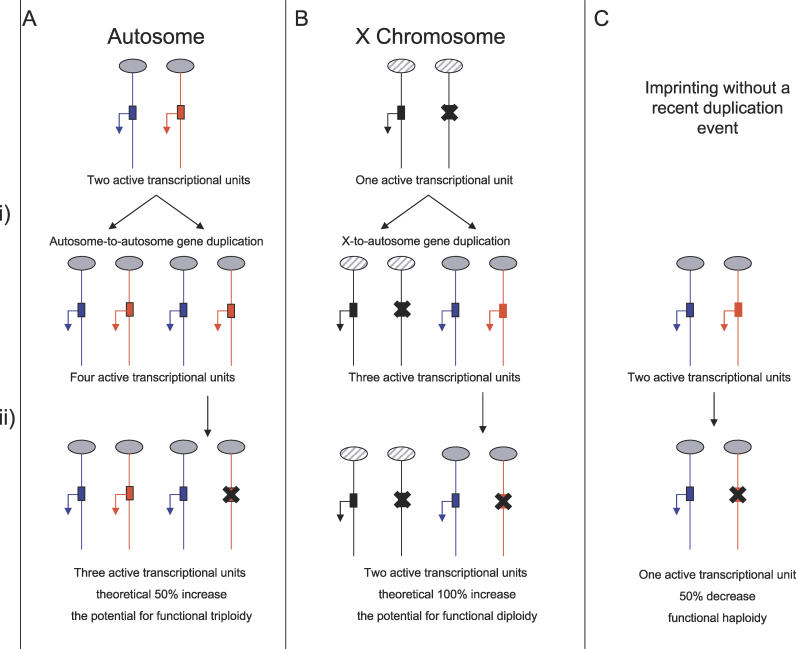
Possible Gene Dosage Scenarios before and after the Acquisition of Imprinting For the two duplication scenarios (A and B), imprinting of the duplicated locus is presumed to arise while functional redundancy exists with the original copy. For simplicity, each example refers to a paternally expressed imprinted gene. Grey ovals represent autosomes, hatched ovals represent the X chromosome, red represents maternally derived chromosomes and alleles, and blue represents paternally derived chromosomes and alleles. (A) A nonimprinted autosomal gene undergoes duplication (i), resulting in a 2-fold (2:4) increase in active gene copy number. Imprinting (ii) reduces this increase to 1.5-fold (2:3). An example is the *Mkrn3* gene on mouse Chromosome 7 [118]. (B) A gene subject to X-inactivation undergoes *trans*-duplication, resulting in a 3-fold (1:3) increase in active gene copy number. Imprinting reduces this increase to 2-fold (1:2). An example is the *U2af1-rs1* locus [58]. (C) A nonimprinted autosomal gene acquires imprinting without undergoing a recent duplication, resulting in a 50% decrease in active gene copy number (2:1). This is likely to be the most common scenario, an example being the *Igf2* locus [95].

Several lines of evidence point to the role of germ-line DNA methylation at ICRs as the primary mark that leads to the establishment of imprinting in the embryo [64–66]. It is therefore instructive to consider what other types of sequence share this property. Differences in gametic methylation patterns are certainly not unique properties of ICRs, as the genome as a whole is more heavily methylated in mature spermatozoa than in oocytes [67]. These differences diminish soon after fertilisation, when the male pronucleus undergoes active demethylation prior to the first zygotic cell division [43]. Paternally methylated ICRs are therefore unusual in their ability to resist this demethylation process [68], although the factors that confer this resistance remain unclear.

Sequence classes such as the primate-specific Alu subfamily of short interspersed elements undergo methylation preferentially in the maternal germ line [69,70]. This differential methylation at Alu elements is relatively short-lived, since they become methylated on both alleles in somatic tissues. Maternally methylated ICRs therefore differ from Alu elements in their ability to resist de novo methylation on the paternal allele during somatic development. All known maternally methylated ICRs have promoter activity on the paternal allele [53], and it is tempting to speculate that transcriptional activity might contribute to this resistance in some way.

Short direct repeat motifs have been identified in proximity to both maternally and paternally methylated ICRs [71], and their potential role in the mechanisms of imprint establishment has been the subject of ongoing debate. Direct repeats at the *Rasgrf1* ICR are required for imprint establishment at this locus [36], whereas the repeat motifs adjacent to the *Igf2* and *Kcnq1* ICRs are dispensable for imprinting at these loci [52,72]. A recent systematic sequence analysis of the CpG islands associated with known imprinted promoters in human and mouse found that they were indeed enriched for direct repeats [73]. Few of these motifs were conserved at the sequence level between the two species, suggesting that secondary DNA structures, rather than specific nucleotide sequences, may provide the target for de novo methylation in the germ line.

While retrogene and transgene insertions can generate alleles that attract methylation [58–60,74], it is also possible that imprinted alleles arise due to germ-line-specific, *trans*-acting factors protecting certain sequences from methylation [44] (Figure 3). Random molecular events such as these could potentially create the allelic raw material on which natural selection can then act.

## What Good Does Imprinting Serve?

Several theories have been put forward to account for the spread of imprinted alleles under the forces of natural selection [7,75,76]. The ovarian time bomb hypothesis states that imprinting occurs to prevent the parthenogenesis of unfertilised oocytes, which can lead to malignant trophoblastic disease [77]. Viable offspring can be generated by the parthenogenesis of oocytes derived from mice carrying a deletion of the *Igf2*/*H19* ICR [78], demonstrating the importance of *Igf2* imprinting in this process. The ovarian time bomb hypothesis predicts that only a small number of genes vital for early embryonic development would be imprinted, and cannot readily explain the involvement of imprinted genes in postnatal traits [14,79]. However, it remains possible that the action of a small number of imprinted genes in preventing parthenogenesis has been advantageous to mammalian populations [80].

The most enthusiastically discussed theory relating to the nature of these forces is the kinship theory, commonly referred to as the conflict hypothesis [81,82]. Put simply, the theory states that when individuals within a litter differ in their degree of relatedness to one another (due to multiple paternities), and parental investment in offspring is unequal (due to maternal provisioning), natural selection would act differently on alleles of maternal and paternal origin in offspring. It is predicted that this manifests as parent-of-origin–specific gene silencing, with paternally derived alleles favouring maternal investment in their own offspring at the expense of simultaneously gestated offspring of different fathers, and with maternally derived alleles serving to maximize the mother's reproductive potential over her reproductive lifespan. The function of many of the first imprinted genes to be discovered provided striking support for these ideas, as the two oppositely imprinted genes *Igf2* and *Igf2r* have strongly opposing roles in fetal growth [3,4].

In mammalian species where offspring continue to acquire resources from the mother postnatally, the conflict hypothesis can account for the involvement of imprinted genes in traits affecting this ongoing acquisition process. Inactivating mutations in the *Gsα* and *Gnasxl* transcripts in the *Gnas* imprinted cluster give rise to opposing postnatal effects on brown fat deposition and metabolic rate [6]. Mice lacking functional copies of the paternally expressed *Gnasxl* gene also demonstrate poor suckling and fail to thrive, consistent with paternally expressed genes acting to promote resource acquisition from the mother [79]. Inactivating mutations in a third gene in the *Gnas* cluster, the maternally expressed *Nesp* transcript encoding the Nesp55 protein, give rise to a phenotype that cannot be directly accounted for by the conflict theory. *Nesp* ^−/+^ individuals show apparently normal embryonic and postnatal development, but exhibit altered reactivity to novel environments [14]. While as-yet-unexplained selective forces may act to maintain the imprinting of this gene, another possibility is that it may be an “innocent bystander” [77], undergoing imprinting serendipitously due to the forces of parental conflict acting on the epigenetic state of neighbouring promoters.

There is little doubt that the phenotypes from mouse knockout models for several imprinted genes provide strong support for the conflict hypothesis in the evolution of parental origin–specific gene expression. However, it seems increasingly unlikely that it can account for the selective forces acting to maintain all imprinted genes [83,84], particularly those expressed primarily in neural tissues and testes such as the genes situated within microimprinted domains (Table 1) ([13,26,85]; A. J. Wood, unpublished data). It should be noted that the conflict hypothesis has been extended to apply to some behavioural traits such as maternal care [86,87]; therefore, expression in neural tissues does not necessarily exclude parental conflict as a factor in the imprinting of such genes. However, the fact that the microimprinted domains are all maternally silenced in the mouse suggests that molecular mechanisms, as well as selective pressures, underlie the allelic expression pattern of these genes.

## Imprinting in the Placenta

As the organ that acts as an interface between mother and fetus, the placenta is predicted to be the site most affected by the forces of the conflict theory. The evolution of the placenta was central to the increased maternal provisioning that distinguishes mammalian development from that of most other vertebrates. The prominent role played by imprinted genes in various aspects of placental physiology [88] suggests that the acquisition of imprinting may have been vital to the evolution of this organ [89,90]. While placentation is often considered a mammalian phenomenon, it is interesting to note that organs of analogous function have evolved independently in fish, reptiles, amphibians, and plants [91–93]. Parent-of-origin–specific gene expression has been demonstrated in the endosperm of angiosperm plants [94], and studies of viviparous nonmammalian vertebrate species will determine whether imprinting is intrinsically linked to the evolution of this organ.

In mammals, the most primitive form of placentation is thought to have evolved over 150 million years ago, after the divergence of early placental mammals (the lineage that would give rise to modern-day marsupials and eutherians) from the egg-laying monotremes and about the same time that the first genes acquired imprinting [95]. Inactivation of one X chromosome is achieved by imprinting in both the placenta and embryo of female (XX) marsupials, suggesting that this was the ancestral mechanism by which sex chromosomal dosage compensation was achieved in early placental mammals [96]. The acquisition of random X-inactivation in the embryonic tissues of eutherian mammals occurred after the marsupial divergence [97], and was apparently accompanied by mechanistic changes in the maintenance of monoallelic silencing [98]. In mice, imprinted X-inactivation and the imprinted silencing of several autosomal genes is mediated by noncoding RNAs in the extraembryonic tissues (reviewed in [99]). Many of these genes are imprinted in the placenta but expressed from both alleles in embryonic cell lineages. Recent work has suggested that several genes that undergo imprinting specifically in the mouse placenta are not imprinted in humans, possibly reflecting the transition from multiple to singleton births reducing the potential for intrabrood competition in primates [100]. Silencing occurs on the paternal allele for the protein-coding genes that are affected in the mouse, and some of the placenta-specific, maternally expressed genes in these regions have potent effects on placental function and embryonic growth [101–103]. It has been suggested that X-inactivation and autosomal imprinting both arose out of a requirement to control the expression of growth-related genes in ancestral placental mammals [90]. The conservation of imprinted X-inactivation between marsupials and eutherians suggests these forces would have been active during the early stages of the mammalian radiation.

The microimprinted domains appear to have arisen specifically in the eutherian lineage and are absent in marsupials ([57,58]; A. J. Wood, unpublished data). As these genes were not in existence during the time period in which placentation first arose, their imprinting cannot have been vital to the early stages of placental evolution. In rodents, genes within the five known microimprinted domains are expressed predominantly in neural tissues and testes ([26,85,104,105]; A. J. Wood, unpublished data), which suggests that tissues other than the placenta may have been primarily influenced by their formation and subsequent imprinting.

## Imprinting of Duplicated Genes

Functional haploidy of imprinted genes is generally viewed as paradoxical, due to the uncovering of recessive mutations that would not occur in the biallelic state of expression (Figure 3). This is true for more ancient vertebrate genes such as *Igf2* and *Impact,* which performed important roles in ancestral vertebrates before acquiring imprinting specifically in the mammalian lineage [106,107]. It is thought that two rounds of whole genome duplication occurred early in vertebrate evolution [108], and it is likely that the *Igf2* gene originated from one of these large-scale events [109]. Imprinting of *Igf2* did not arise until the emergence of placental mammals, after their divergence from egg-laying ancestors [95,106]. Although *Igf2* is derived from a gene duplication event, two key factors distinguish this situation from that of imprinted retrogenes. First, the transcriptional profile of the gene duplicate would be expected to mimic that of the ancestral gene, as the *cis*-acting regulatory elements would have been duplicated in addition to the transcribed sequence. In contrast, the expression pattern of novel retrogenes is much more difficult to predict, as factors associated with the site of integration affect the expression pattern of the gene duplicate [110]. Second, the duplication of *Igf2* is unlikely to have been directly linked to the subsequent establishment of imprinting at the locus, as several hundred million years elapsed between the two evolutionary events. It is likely that the two genes would have undergone functional divergence during this time period to fulfill distinct roles.

Genes that originated from retrotransposition events that occurred later in mammalian evolution are likely to have undergone imprinting soon after their formation, and would therefore have shown functional redundancy with the parent copy in tissues in which both are expressed. For these genes, monoallelic silencing may have been in the immediate interests of the host. Imprinting would serve to reduce the potential transcriptional dosage imbalance resulting from the increase in copy number, while maintaining one active allele with the potential to evolve novel functions.

Compared with the autosomes, the X chromosome has generated a disproportionately high number of functional retrogenes in mammalian species [111], at least three of which undergo imprinting at their autosomal location in the mouse genome ([13,26,58]; A. J. Wood, unpublished data). Figure 4 illustrates how the process of X-inactivation may have impacted the active gene–dosage imbalance associated with these events. How this theory would relate to the total transcriptional output of the gene family is unclear, due to the global upregulation of the single active X chromosome relative to autosomes [112].

Gene duplications have occurred throughout the course of evolution, and a weakness of the current argument is that similar epigenetic effects have not been observed in nonmammalian vertebrates. However, mammals differ from other vertebrates in the epigenetic mechanisms that are employed to defend the genome against transposable elements, and it has been proposed that imprinting is mediated by mechanisms that were originally used for this purpose [113,114]. These ideas are commonly referred to as the host–defense theory, and may be particularly applicable to retrogenes situated within microimprinted domains [13,58], where the retrotransposed sequences themselves are targeted for methylation in the maternal germ line. By combining the host–defense theory [113,114] with transcriptional dosage imbalance resulting from gene duplication [115], it may be possible to account both for molecular events by which imprinted alleles can be generated, and for the selective forces that might favour their spread in a population.

## Conclusions

To gain a comprehensive understanding of the function of imprinting in mammals, it is first necessary to define the full extent of this phenomenon and the physiological processes that it affects. Genome-wide screens and knockout studies of individual genes in the mouse will be key tools in addressing this goal, and it will be interesting to see the extent to which existing hypotheses are supported. Much of our current knowledge of imprinting is derived from the laboratory mouse, and it is of note that recent work in humans has highlighted some key differences, particularly in the placenta [100]. Understanding imprinting in outbred populations will determine the extent to which these ideas can be extrapolated to nondomesticated mammalian species.

## Supporting Information

### Accession Numbers

The GenBank (http://www.ncbi.nlm.nih.gov/Genbank/index.html) accession numbers for the genes listed in Table 1 are as follows: *Inpp5f_v2* (DQ648020), *Nap1l5* (NM_021432), *Nnat* (NM_010923), *Peg13* (AY151253), and *U2af1-rs1* (NM_011663).

## Abbreviations

CTCF: CCCTC-binding factor
ICR: imprinting control region
